# DLL3-directed immune redirection in small-cell lung cancer: a lineage-defined vulnerability that doubles as an escape route

**DOI:** 10.3389/fimmu.2026.1901163

**Published:** 2026-07-28

**Authors:** Xitan Wang, Fuli Wang, Wei Tian

**Affiliations:** Department of Oncology, Zibo Central Hospital, Zibo, China

**Keywords:** acquired resistance, ASCL1, bispecific T-cell engager, DLL3, neuroendocrine lineage plasticity, small-cell lung cancer, tarlatamab, T-cell exhaustion

## Abstract

Delta-like ligand 3 (DLL3) is the first tumor cell surface antigen in small cell lung cancer (SCLC) to support an approved T-cell-redirecting therapy with demonstrated survival benefit, namely the DLL3×CD3 bispecific T-cell engager tarlatamab. DLL3-directed agents are usually surveyed by platform. Here, we argue instead that DLL3 is best understood not as a static surface target but as the surface readout of a plastic, ASCL1-associated neuroendocrine lineage state. DLL3-directed immune redirection works by holding two intrinsically unstable systems in transient alignment: a lineage-defined antigen state on the tumor side and an activatable but exhaustible T cell effector system on the host side. This framework helps explain why the same antigen has produced different clinical outcomes across therapeutic platforms. The failure of rovalpituzumab tesirine does not establish that DLL3 is intrinsically unsuitable for payload delivery; rather, it highlights the construct-specific challenge of achieving a sufficient therapeutic index with a PBD-based antibody-drug conjugate. Tarlatamab clinically validates a distinct use of DLL3—as a surface recognition tag for T-cell redirection—while leaving open the possibility that redesigned DLL3-directed antibody-drug conjugates may succeed. That recruitment makes therapeutic success contingent on a host effector system the drug does not fully control, and resistance correspondingly emerges along two axes. In antigen-side escape, therapeutic pressure may select for, or in some contexts drive, a DLL3-low or non-neuroendocrine state. In execution-side escape, pre-existing T-cell dysfunction may limit primary efficacy, whereas repeated redirected activation may deepen exhaustion within a suppressive niche. The lineage-defined vulnerability that the therapy exploits can thus double as the route of escape. We propose that next-generation strategies should be organized by the axis they are designed to preserve, and that the operative clinical question should shift from baseline DLL3 positivity toward the durability of antigen availability and effector function.

## 1 Introduction: rethinking DLL3 beyond a static surface target

Small cell lung cancer (SCLC) is among the most aggressive solid tumors. It is a neuroendocrine malignancy that disseminates early, responds briskly to first-line platinum-based chemotherapy, and then relapses almost universally, with survival in the relapsed setting historically measured in months. Accounting for roughly 15% of lung cancers, SCLC is biologically defined by near-universal loss of TP53 and RB1, neuroendocrine differentiation, and rapid acquired resistance. These features long left the disease with few actionable surface targets, and SCLC largely resisted the targeted therapy revolution that reshaped non-small cell lung cancer while effective second-line options remained scarce (1, 2). Against this long stagnation, delta-like ligand 3 (DLL3) has become the first tumor cell surface antigen in SCLC to support an approved T-cell redirecting therapy with a demonstrated survival benefit: the DLL3×CD3 bispecific T-cell engager tarlatamab (3–5). Over roughly a decade of development, DLL3 has thereby moved from a candidate antigen to a clinically validated vulnerability.

The field is usually surveyed through the lens of modality. Existing reviews catalogue DLL3-directed agents by platform, including antibody drug conjugates, bispecific T-cell engagers, chimeric antigen receptor cells, and radioimmunotherapy, and track their respective trials (6–8). This organization is useful, but it can leave DLL3 looking like a fixed surface address and the clinical story like a succession of platforms. This review takes a different view. DLL3 is better understood not as a static surface label but as the surface readout of a particular neuroendocrine lineage state, and DLL3-directed immune redirection works by exploiting that state rather than a free-standing antigen. The value of this lens is that it explains therapeutic success and failure with a single logic: the same coupling to a specific and plastic lineage identity that makes DLL3 a vulnerability can also turn it into a route of escape.

**Figure 1:**
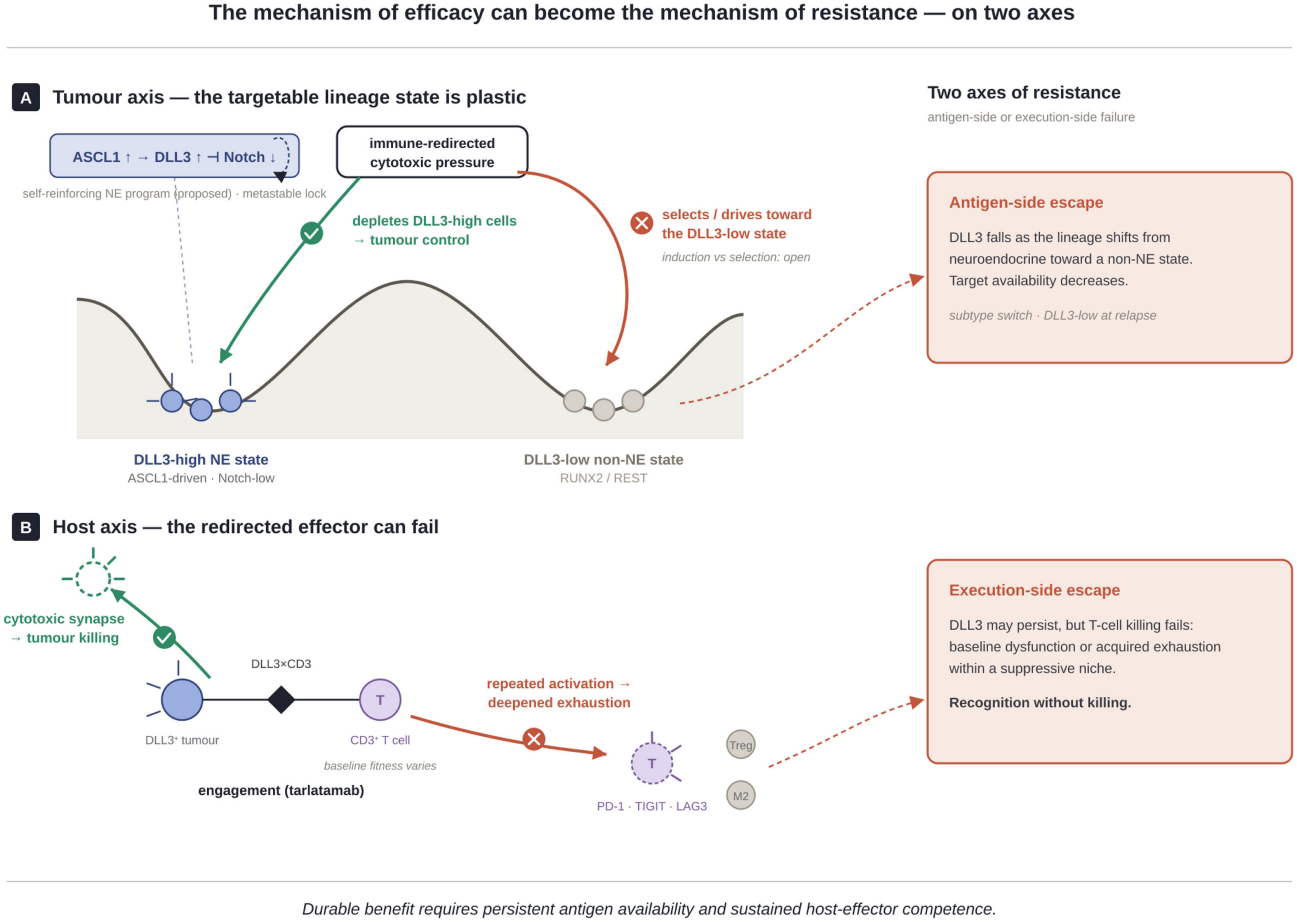
DLL3-directed immune redirection in SCLC: resistance can emerge along tumor and host axes. **(A)** Tumor axis. A proposed self-reinforcing ASCL1–DLL3–Notch program helps support a metastable DLL3-high neuroendocrine state, rendering DLL3 frequently expressed and therapeutically accessible. DLL3-directed cytotoxic pressure preferentially depletes DLL3-high cells but may select for, and in some contexts promote, DLL3-low non-neuroendocrine states in which target availability decreases. Whether this reflects selection of pre-existing populations or treatment-induced lineage transition remains unresolved. **(B)** Host axis. The DLL3×CD3 bispecific bridges DLL3-positive tumor cells and CD3-positive T cells to produce cytotoxic killing. Pre-existing T-cell dysfunction may blunt the initial response, whereas repeated redirected activation can deepen exhaustion during treatment, marked by PD-1, TIGIT, and LAG-3 within an immunosuppressive niche containing regulatory T cells, tumor-associated macrophages, and myeloid-derived suppressor cells. Durable benefit therefore requires persistent antigen availability and sustained host-effector competence.

Stated more precisely, DLL3-directed immune redirection succeeds by holding two intrinsically unstable systems in transient alignment: a plastic, lineage-defined antigen state on the tumor side, and an activatable but exhaustible T-cell effector system on the host side. Durable benefit requires both systems to hold at the same time (**Figure 1**). Resistance, on this account, is not a separate event grafted onto the therapy, but the same mechanism running forward under therapeutic pressure. The cytotoxic pressure that clears DLL3-high neuroendocrine cells may select for, and in some contexts may actively drive, DLL3-low states that escape it. At the same time, the sustained T-cell activation that produces tumor killing can also drive exhaustion and suppressive microenvironmental remodeling that eventually limits killing. This review develops the argument in sequence: the lineage origin of the DLL3 antigen and its instability; the platform-specific requirements revealed by the divergent clinical trajectories of Rova-T and tarlatamab; the clinical validation of redirection and the cost coupled to its mechanism; and finally, resistance along two axes, namely collapse of the antigen state and failure of immune execution, together with strategies directed at each.

## 2 The lineage origin of DLL3 vulnerability: a plastic neuroendocrine state

The first of the two systems on which DLL3-directed therapy depends is the tumor’s own antigenic state, and that state originates in the lineage biology of SCLC. SCLC is predominantly a neuroendocrine malignancy, and transcription-factor-based subtyping assigns a substantial, often dominant fraction of tumors, approximately 60% in most series, to the ASCL1-driven neuroendocrine subtype, SCLC-A, as distinct from the NEUROD1-, POU2F3-, and YAP1/inflamed-defined subtypes (9, 10). ASCL1 is a master regulator of this neuroendocrine program, and DLL3 has been identified as an ASCL1-bound and ASCL1-associated transcriptional output by chromatin immunoprecipitation and related regulatory studies. DLL3 expression therefore tracks the ASCL1-high neuroendocrine state rather than existing independently of it (11–13). Genomically, SCLC is defined by near-universal inactivation of TP53 and RB1 and, of particular relevance here, by recurrent inactivating mutations in NOTCH-family genes together with generally low Notch-pathway activity (14). Because canonical Notch activation suppresses the neuroendocrine program, in part through HES1-mediated repression of ASCL1, the neuroendocrine identity of SCLC is characteristically a Notch-low state. It is in this low-Notch, ASCL1-high context that DLL3 is expressed.

DLL3 occupies an unusual position within this circuitry. It is a Notch ligand, but an atypical and largely inhibitory one. Compared with canonical activating Notch ligands, DLL3 is enriched intracellularly and acts predominantly in cis, where it sequesters Notch receptors in the Golgi and thereby dampens rather than activates Notch signaling. Nonetheless, DLL3 is also presented on the tumor-cell surface at levels sufficient to support antibody-based targeting (15–17). As both an ASCL1-associated transcriptional output and an inhibitory Notch ligand, DLL3 may therefore not only mark the Notch-low neuroendocrine state but also contribute to maintaining it, forming a reinforcing relationship rather than serving as a passive readout (16). This interpretation should be regarded as a mechanistic hypothesis rather than an established regulatory circuit. The evidence firmly supports DLL3 as an ASCL1-associated output and an inhibitory Notch ligand, and is consistent with, but does not prove, a self-stabilizing loop. What is clinically concrete is the consequence. At diagnosis, many treatment-naïve SCLC-A tumors occupy a relatively self-consistent ASCL1-high, Notch-low, DLL3-high configuration. DLL3 is expressed in the majority of SCLC, on the order of 80% or more of tumors, and frequently at high levels, while among normal adult tissues it is largely restricted to a few cell types (17, 18). This combination of high, tumor-enriched surface expression and low normal-tissue expression is precisely what makes DLL3 a targetable antigen and opens the therapeutic window exploited by the therapies discussed below.

That configuration, however, is metastable rather than fixed, and this instability, rather than the apparently stable state, is what the rest of the review turns on. The same Notch axis that is suppressed in the neuroendocrine state can be reactivated, and Notch signaling has been shown to drive a transition from a neuroendocrine to a non-neuroendocrine phenotype. This process generates intratumoral heterogeneity and a distinct non-neuroendocrine population that is typically slower-growing and relatively chemoresistant (19, 20). In such non-neuroendocrine states, the ASCL1 program is downregulated, and because DLL3 is an ASCL1-associated transcriptional output, its expression typically declines in parallel, becoming lower or more heterogeneous. Critically, this plasticity is not exclusively Notch-dependent. A shift toward the non-neuroendocrine state can occur even in the absence of NOTCH, through alternative routes such as a RUNX2/REST-associated program (21). Notch is therefore the best-characterized regulator of the neuroendocrine-to-non-neuroendocrine axis, but it is one regulator of a broader lineage plasticity rather than its sole determinant. The operative variable is the lineage state itself, not any single pathway. This distinction matters because it implies that the DLL3 antigen can be lost through more than one molecular route.

The unifying consequence for DLL3 is the central claim of this section: DLL3 is best understood not as a static surface label but as the surface readout of a particular and reversible lineage identity. Its expression is accordingly neither uniform nor permanent. Across SCLC and the wider family of pulmonary and extrapulmonary neuroendocrine neoplasms, DLL3 expression is prevalent but variable. It differs between tumors and tracks the degree of neuroendocrine differentiation. Although DLL3 expression is often relatively uniform within an individual treatment-naïve tumor, it is neither uniform across tumors nor stable over the disease course (18, 22). Such variation should not be dismissed as measurement noise, but should instead be understood as the surface manifestation of variability in the underlying lineage state. The instability is temporal as well as spatial. Circulating tumor-cell analyses can track DLL3-bearing tumor cells in the blood, and pretreatment CTC measures have been associated with subsequent response to DLL3-directed therapy. This supports viewing DLL3 not only as a fixed tissue stain but also as a dynamic, systemic antigen state (23). Taken together, these properties define the kind of target DLL3 actually is: tumor-enriched and abundant enough at diagnosis to constitute a genuine therapeutic window, yet tied to a plastic neuroendocrine identity, so that antigen availability depends on persistence of the state that produces it. The same plasticity that exposes DLL3 also implies that it can be lost, a possibility we return to in the context of resistance. It also sharpens the immediate question: given a conditionally expressed, lineage-defined surface antigen, how should it be exploited therapeutically? Two DLL3-targeting strategies answered that question in opposite ways, with instructive consequences.

## 3 Platform-specific use of DLL3: from Rova-T to T-cell redirection

DLL3 is therefore not simply a surface antigen to be bound, but a conditionally available, lineage-defined target whose therapeutic use depends on how that surface presence is converted into tumor-cell death (11–13, 17, 19–21). Rova-T and tarlatamab illustrate two distinct ways of exploiting DLL3, but their clinical outcomes should not be interpreted as a controlled comparison of antigen escape or as evidence that DLL3 is intrinsically unsuitable for payload delivery. The published Rova-T trials did not include longitudinal antigen analyses sufficient to establish whether treatment selected for DLL3-negative disease (24–26). Their contrast is therefore most informative about the different platform-specific requirements imposed by an antibody-drug conjugate and a T-cell engager. In the former, the antibody must bind, internalize, traffic intracellularly, and release a sufficiently potent payload while maintaining an acceptable systemic therapeutic index (17, 24–26). In the latter, DLL3 functions as a surface recognition tag that nucleates a productive cytolytic synapse with a recruited T cell (27).

Rovalpituzumab tesirine (Rova-T) embodied the antibody-drug conjugate strategy: an anti-DLL3 antibody conjugated through a cleavable linker to a pyrrolobenzodiazepine (PBD) dimer DNA-crosslinking payload (17, 24). Encouraging first-in-human activity, particularly in DLL3-high tumors, generated early enthusiasm (24), but randomized trials did not sustain this signal. In the phase III TAHOE study, Rova-T produced inferior overall survival to topotecan and the trial was halted early; the MERU maintenance study likewise failed (25, 26). Rova-T also produced a characteristic non-hematological toxicity profile, including pleural and pericardial effusions, photosensitivity, and peripheral edema, which, together with its limited efficacy in randomized trials, indicated an unfavorable clinical therapeutic index for the PBD-containing construct (24–26). Preclinically, however, SC16LD6.5 bound DLL3 and delivered its payload with sufficient activity to produce durable regression in DLL3-expressing SCLC and large-cell neuroendocrine carcinoma xenografts and to deplete tumor-initiating cells (17). These findings support the feasibility of DLL3-dependent internalization and payload delivery in preclinical model systems (17). The later clinical trials did not directly quantify intratumoral antibody internalization, linker cleavage, or intracellular payload exposure in patients and therefore cannot exclude heterogeneous or insufficient delivery in clinical tumors (24–26). Nor did they report longitudinal DLL3 measurements capable of demonstrating selection for DLL3-negative disease (24–26). Taken together, the available evidence supports the conclusion that this PBD-based antibody-drug conjugate failed to achieve an adequate clinical therapeutic index, owing to payload-associated toxicity and potentially other delivery-related limitations, rather than demonstrating that DLL3 is intrinsically unsuitable for antibody-drug-conjugate-mediated delivery (17, 24–26). These limitations leave open the possibility that redesigned DLL3-directed antibody-drug conjugates incorporating safer payloads or optimized linker and conjugation properties could succeed.

Tarlatamab exploits the same antigen without requiring antibody internalization or intracellular payload release. It is a half-life-extended DLL3×CD3 bispecific T-cell engager that carries no cytotoxic payload (27). Its proximal function is to bring a DLL3-positive tumor cell and a CD3-positive T cell into a synapse, triggering MHC-independent redirected polyclonal T-cell activation and tumor-cell lysis (27). This platform removes the requirements for antibody internalization, intracellular trafficking, linker cleavage, and payload release after target binding (6, 8, 17, 27). It instead introduces dependencies on accessible DLL3 expression, productive tumor-T-cell engagement, baseline T-cell fitness, tissue access, and local immunosuppression (27–30). After synapse formation, tumor killing can be amplified through recruited effector cells and is not determined solely by the amount of antigen-bound therapeutic molecule (6, 8, 27). Tarlatamab therefore validates DLL3 as a clinically actionable recognition target, but does not establish that recognition is the antigen’s only therapeutically viable use or that future DLL3-directed antibody-drug conjugates cannot succeed (3, 24–27).

By using DLL3 as a recruitment signal, T-cell redirection makes therapeutic success contingent on a system the drug recruits but does not fully control: the T-cell effector compartment. Recognition remains necessary, but it is no longer sufficient. Between antigen binding and tumor-cell death lie baseline T-cell fitness, productive tumor-T-cell engagement, the immunosuppressive microenvironment, and the durability of the effector response (27–31). Together with antigen persistence on the tumor side, effector execution on the host side defines the second axis of therapeutic vulnerability. This logic leads directly to the clinical question addressed by tarlatamab: whether DLL3 recognition can generate meaningful tumor regression (3, 27). It also raises the question of what acute and chronic costs follow from transferring tumor killing to an activatable but potentially dysfunctional T-cell compartment (28, 29, 31–34).

## 4 Clinical validation and the cost of redirected T cells

For two decades, relapsed SCLC exemplified the limits of available therapy: after platinum-based induction, second-line treatment was dominated by topotecan-class chemotherapy, with response durations measured in weeks and no agent convincingly extending survival (1, 2). Against this backdrop, the clinical results with tarlatamab represent the first clearly survival-extending advance to be validated in the relapsed setting. In the phase II DeLLphi-301 study, tarlatamab at 10 mg every two weeks produced an objective response in 40% of heavily pretreated patients, with a 97.5% CI of 29 to 52, far above the historical benchmark of roughly 15% in this setting. The responses were also durable, with 59% of responders maintaining a response for at least six months (27, 35). These results provided the first clinical proof that the recognition-tag logic was operational in SCLC: engaging DLL3 on the tumor surface and CD3 on the T cell could drive clinically meaningful and durable tumor regression in patients, not only in models.

DeLLphi-304 converted this signal into a validated standard. In a randomized phase III trial against investigator’s-choice chemotherapy, tarlatamab significantly prolonged overall survival, with a median overall survival of 13.6 versus 8.3 months, a hazard ratio of 0.60, and P<0.001. This benefit was accompanied by concordant progression-free survival and patient-reported symptom improvements. Unusually for an active agent in this disease, tarlatamab also produced a lower rate of high-grade adverse events than chemotherapy (3). On this basis, DLL3-directed immune redirection has moved from a promising target concept to a an approved, survival-extending therapy (4, 5, 36). The antigen is therefore not merely bindable but clinically actionable: in the post-platinum setting, DLL3 expression is sufficiently exposed and therapeutically exploitable to support a survival benefit, even if it is not uniformly predictive at the individual-patient level (23, 37). A corollary of this framing concerns the host and tumor features that might be expected to predict benefit. In contrast to checkpoint blockade, where mutational burden and smoking-associated neoantigens may contribute to response patterns, redirection is MHC- and neoantigen-independent, so a smoking- or mutational-burden-based logic need not transfer. Consistent with this, early correlative analyses have not linked tumor mutational burden to tarlatamab response, which instead tracks DLL3 expression, ASCL1-driven lineage, and immune activation (37); the operative predictors are again antigen state and effector context rather than mutational history. Ethnicity is similarly underexplored: although a dedicated Asian subgroup analysis of DeLLphi-301 is available (35), whether ethnic or pharmacogenomic background modifies efficacy, or the incidence of immune-mediated toxicity such as CRS, remains unaddressed by adequately powered comparisons. These gaps reinforce rather than complicate the review’s central point—that the variables governing DLL3-directed redirection are the durability of antigen availability and of effector function, not the baseline categorical features that have guided other immunotherapies.

That benefit, however, is inseparable from a characteristic cost, and the cost is on-mechanism. Because tarlatamab acts by forcing a synapse between tumor cell and T cell and driving polyclonal T-cell activation, its dominant toxicities are the predictable consequences of that activation: cytokine release syndrome (CRS), the most frequent adverse event, largely low-grade and concentrated around the priming and first full doses, together with immune effector cell-associated neurotoxicity (ICANS) and related neurologic events (3, 27, 32). The mitigations are themselves mechanistic and impose a system-level burden: step-up priming dosing to blunt the initial activation peak, early-cycle monitoring, and supportive care with hydration, tocilizumab, and corticosteroids (32, 38). The magnitude of that burden is an active question rather than a fixed property. A DeLLphi-300 substudy suggests that, in selected patients, abbreviated outpatient monitoring may be feasible and could reduce reliance on prolonged inpatient observation (39). Real-world series, in turn, extend the efficacy signal to trial-ineligible populations, including patients with untreated brain metastases, poorer performance status, organ dysfunction, or other comorbidities (40–43). These studies also indicate that CRS and ICANS rates, monitoring needs, and delivery logistics may differ from the controlled-trial setting, particularly in patients with CNS involvement or medical fragility (40, 43). Reports of rapid intracranial responses are especially notable because CNS involvement is common in SCLC and has historically been under-represented in trials, making intracranial activity an important but insufficiently defined aspect of DLL3-directed immune redirection (44).

The unifying observation is that the same T-cell activation that produces the survival benefit also produces the toxicity and monitoring burden. Efficacy and its principal acute cost are therefore two readouts of a single mechanism, rather than independent properties that can be optimized separately. This is where the therapeutic trade-off of DLL3-directed immune redirection becomes clinically visible. If the mechanism’s benefit and acute cost are inseparable, it is reasonable to ask whether the same mechanism might also contribute to eventual treatment failure. Acute toxicity is the early and often manageable face of a mechanism-linked cost. Resistance may represent its chronic and selective face.

## 5 Resistance: when the mechanism of action becomes the mechanism of escape

DLL3-directed redirection requires two conditions to hold at the same time: a tumor that presents the lineage-defined antigen, and a host T-cell compartment able to execute the killing it is recruited to perform (27). Resistance can therefore be understood as a failure of this alignment, with failure occurring along the same two axes on which the therapy depends. Emerging correlative studies, although still small and largely retrospective, support this two-axis structure with notable directness. In one circulating-tumor-cell series, acquired resistance was accompanied in some patients by loss of DLL3 with persistence of other neuroendocrine epitopes, and in others by retention of DLL3 together with systemic markers of T-cell dysfunction (23). These findings point to two separable forms of resistance: antigen-side escape and execution-side escape. Because the same report shows that these modes can occur separately, they are best treated as distinct axes rather than as a single pathway (**Table 1**).

**Table 1:** Dual-axis resistance framework for DLL3-directed immune redirection in SCLC.

Comparison dimension	Antigen-side escape	Execution-side escape
What fails	The target. DLL3 is lost or reduced as the cell changes lineage identity.	The effector. DLL3 persists, but the recruited T cell can no longer kill.
Core mechanism	Lineage shift away from the ASCL1-high/DLL3-high neuroendocrine state toward DLL3-low or alternative-subtype states, including NEUROD1-high or YAP1-associated programs. DLL3, an ASCL1-associated transcriptional output, declines with it.	Pre-existing exhausted or dysfunctional T-cell states may limit immediate T-cell-engager responsiveness, while repeated redirected activation can further drive progressive exhaustion within an immunosuppressive or T-cell-excluded niche containing regulatory T cells and suppressive myeloid cells.
Link to mechanism of action	Cytotoxic pressure falls selectively on engageable DLL3-high cells, enriching DLL3-low populations that are less efficiently engaged or eliminated.	Execution can fail before dosing because of limited baseline T-cell fitness or emerge during treatment as repeated activation deepens dysfunction. The same redirection that produces killing can therefore expose or intensify an effector limitation.
Causal certainty	Open. Selection versus induction of lineage shift remains unresolved. The most direct evidence to date favors selection against the ASCL1 lineage.	Mechanistically grounded across T-cell-engaging therapies, but the relative contributions of baseline dysfunction, treatment-emergent exhaustion, and microenvironmental suppression remain to be defined in tarlatamab-treated SCLC.
Supporting evidence	Subtype change at resistance (45); transcriptomic correlates of response and resistance (28, 37); CTC subset losing DLL3 but retaining other neuroendocrine epitopes (23); paired-biopsy phenotype shift with reduced neuroendocrine differentiation (29).	CTC subset retaining DLL3 with emergent systemic T-cell dysfunction (23); paired biopsies showing exhaustion and a suppressive niche not explained by antigen loss (29); resistant tumors enriched for regulatory/exhausted T-cell programs (28); cross-platform evidence for baseline T-cell-state dependence and continuous-exposure exhaustion (30, 31); myeloid inflammatory and suppressive mechanisms supporting the acute-to-chronic bridge (33, 34, 47, 48).
Monitoring readouts	Serial CTC-DLL3 assessment (23); ctDNA for tumor-burden dynamics (42); longitudinal single-cell or spatial profiling to distinguish selection from induction (46).	Baseline and longitudinal T-cell phenotyping for exhaustion and functional markers; microenvironmental immune profiling; correlates of persistent effector function.
Repair strategies	Co-target a second SCLC-enriched antigen such as B7-H3, SEZ6, or TROP2 (6–8); add antigen-agnostic redundancy; if induction dominates, consider epigenetic or lineage-state co-targeting, including EZH2, LSD1, or Aurora kinase-directed approaches (52–54).	DLL3-targeted CD3/CD137 costimulation (50); PD-(L)1 combination (51); DLL3-directed CAR-T platforms, including allogeneic and IL-18-armored designs (55–57); niche remodeling.

Antigen-side and execution-side escape are two faces of a single therapeutic logic: the lineage-defined vulnerability that the therapy exploits can double as the route of escape. They are not interchangeable for treatment and may co-occur. Reference numbers follow the manuscript citation order. The antigen axis is treated as causally open because selection versus induction remains unresolved. The execution axis includes both baseline T-cell dysfunction and treatment-emergent exhaustion; its general biological basis is supported across T-cell-engaging platforms, but the relative contributions of these components in tarlatamab-treated SCLC remain unresolved. CTC, circulating tumor cell; ctDNA, circulating tumor DNA.

### 5.1 Antigen-side escape: pressure toward DLL3-low or non-neuroendocrine states

The first axis is the loss of the target itself. Because DLL3 is the surface readout of an ASCL1-driven neuroendocrine identity rather than a free-standing antigen, the antigen can be lost not only by selective downregulation but also through a coordinated change in tumor-cell identity. This is the resistance prediction made by the lineage view and missed by a static-antigen view: target loss should travel with a shift in lineage state, rather than occur as isolated antigen disappearance. The available data, although still preliminary, are consistent with this interpretation. Analyses of tumors progressing on tarlatamab describe a decline in DLL3 expression accompanied by lineage-state reprogramming, including loss of the ASCL1-high neuroendocrine program and emergence of non-neuroendocrine or alternative transcription-factor states, such as YAP1-associated or NEUROD1-high programs, rather than antigen loss in isolation (28, 37, 45). This pattern has been reported in a small resistant series, in a transcriptomic analysis of paired pre- and post-treatment tumors, and, most directly, in a study combining longitudinal plasma transcriptional profiling with a syngeneic mouse model (28, 37, 45). A parallel observation in paired biopsies is a phenotypic shift toward cells with abundant cytoplasm and reduced synaptophysin, consistent with attenuated neuroendocrine differentiation (29). Notably, similar lineage transitions have been described as resistance routes after chemotherapy and checkpoint blockade, suggesting that neuroendocrine-to-non-neuroendocrine plasticity is a convergent escape pathway rather than one specific to T-cell redirection.

The therapy’s mechanism and its failure converge at this point, but the limits of current evidence must be stated plainly. The cytotoxic pressure of redirection falls selectively on DLL3-high neuroendocrine cells that can be effectively engaged, and would be expected to enrich DLL3-low or non-neuroendocrine populations that are less efficiently engaged or eliminated (28, 37, 45). Whether redirection selects for pre-existing DLL3-low clones or actively drives lineage transition remains unresolved, and the distinction matters therapeutically. Selection would favor strategies that pre-empt or co-target resistant populations, whereas induction would additionally motivate attempts to modulate lineage plasticity itself. The most direct recent evidence describes therapeutic selection for a NEUROD1-high, DLL3-low state under DLL3-directed T-cell engagement, consistent with selective pressure against the ASCL1-driven lineage rather than *de novo* induction (28). However, this conclusion rests on preprint-level evidence and does not exclude an inductive contribution. Because SCLC lineage plasticity is intrinsic and not exclusively Notch-dependent, antigen-directed therapy operates on a moving target (19–21). The dominant direction of causation, and the frequency with which selection versus induction operates, remain open questions rather than settled facts. Resolving this question will require longitudinal, high-resolution sampling rather than endpoint comparisons alone. Serial biopsies analyzed by single-cell RNA sequencing, circulating tumor-cell profiling, and spatial transcriptomics could distinguish expansion of pre-existing DLL3-low subclones from therapy-induced state transitions, while preserving the relationship between lineage programs and immune niches (46). Such studies would move the antigen-side escape model from a plausible lineage framework toward a directly testable evolutionary model.

### 5.2 Execution-side escape: T-cell exhaustion and suppressive niches

The second axis fails with the antigen still present. Because redirection delegates killing to the host T cell, the response can collapse even when DLL3 remains expressed if the recruited effector cannot execute (23, 27, 29). Execution-side failure may be present before treatment or emerge during therapy (30, 31). Studies of other CD3-directed bispecifics indicate that the pre-existing T-cell landscape can influence responsiveness, with exhausted-like or dysfunctional states associated with inferior activity (30). This cross-platform evidence does not establish the same relationship in tarlatamab-treated SCLC, but it supports the possibility that baseline T-cell fitness contributes to primary resistance (30). During treatment, repeated engager-mediated stimulation may further deepen dysfunction; continuous bispecific exposure has produced progressive loss of cytotoxic function and an exhaustion-associated transcriptional program in experimental systems (31).

In tarlatamab-treated SCLC, the available evidence remains largely correlative but supports execution-side failure as a distinct resistance mode. In a subset of patients, DLL3 was retained on circulating tumor cells at progression while systemic markers of T-cell dysfunction emerged (23). Paired-biopsy analysis of progression on tarlatamab combined with PD-1 blockade also implicated T-cell exhaustion and a suppressive tumor microenvironment that could not be explained by antigen loss alone (29). Consistently, an integrated plasma and model study identified a separate group of resistant tumors enriched for regulatory and exhausted T-cell programs (28). These observations suggest that pre-existing dysfunction, treatment-emergent exhaustion, and microenvironmental suppression may converge on the same endpoint: recognition without effective killing.

The myeloid compartment provides an important bridge between the acute and chronic faces of this mechanism. During early immune redirection, activated T cells can engage bystander innate immune cells, particularly monocytes and macrophages, amplifying IL-6- and IL-1-dominated inflammatory circuits that contribute to cytokine release syndrome and may intersect with ICANS-associated inflammation (33, 34). Over a longer time scale, the same compartment can become a suppressive niche: tumor-associated macrophages and myeloid-derived suppressor cells restrain cytotoxic T-cell function through cytokine, metabolic, and checkpoint-mediated mechanisms (47, 48). Thus, myeloid biology can link the acute cost of redirected activation to chronic failure of immune execution.

Although the proximal cytotoxic action of DLL3×CD3 engagement is independent of tumor-cell MHC recognition (27), downstream immunity may not remain entirely MHC-independent. Preclinical T-cell-engager studies suggest that tumor-cell lysis and local inflammation can promote the release and presentation of additional tumor antigens and thereby recruit endogenous MHC-restricted T-cell responses (49). A secondary CD8-positive T-cell response against antigens beyond the originally targeted antigen has been demonstrated in a preclinical BiTE system (49), raising the possibility of epitope spreading that could partly buffer antigen heterogeneity. This mechanism has not yet been established for tarlatamab in SCLC and should therefore be regarded as a plausible secondary contribution rather than a demonstrated clinical effect.

Many execution-side determinants discussed here are not unique to DLL3. Baseline T-cell fitness, immune-cell infiltration, chronic stimulation, checkpoint feedback, and suppressive myeloid or regulatory-cell niches are general constraints on CD3-engaging therapies across disease settings (30, 31, 47, 48, 50, 51). What is relatively distinctive in DLL3-directed SCLC therapy is the coupling of target availability to a plastic ASCL1-associated neuroendocrine lineage state (11–13, 19–21, 28, 37, 45). The execution axis is therefore broadly applicable to T-cell engagers, whereas the biological architecture of the antigen axis is target- and tumor-context dependent.

### 5.3 Closing the loop

Read together, the two axes support the framing in the title (**Figure 1**). Under therapeutic pressure, the lineage-defined antigen state may shift toward a DLL3-low or non-neuroendocrine identity, while immune execution may fail because the recruited T-cell compartment is constrained at baseline or becomes exhausted during repeated activation. These modes are distinct but may co-occur, including progression with both reduced DLL3 and effector dysfunction (29). Because target loss and effector failure require different countermeasures, emerging strategies should be judged by whether they preserve antigen availability, effector function, or both.

## 6 Resistance-adaptive strategies and conclusion: repairing antigen recognition and immune execution

If resistance arises along two distinct axes, rational next-generation strategies should be evaluated by which axis they are designed to preserve, rather than by molecular platform alone. A more potent engager does not address antigen loss, and a second antigen does not address T-cell exhaustion. Sorting emerging approaches in this way clarifies what each strategy can and cannot be expected to achieve, and highlights where both axes must be addressed together. With few exceptions, these approaches remain preclinical or early-phase. They are therefore presented here as a mechanistic logic rather than as validated interventions.

### 6.1 Holding the antigen axis: monitoring and diversifying the target

The antigen axis fails when the lineage state that presents DLL3 moves. Two complementary responses follow. The first is to stop treating DLL3 as a fixed pretreatment property and instead monitor it as a dynamic state. Serial circulating tumor-cell assays could track DLL3 availability, whereas circulating tumor DNA may complement these measurements by monitoring tumor-burden dynamics; longitudinal tissue or single-cell profiling would be required to resolve lineage evolution (23, 42, 46). Together, these tools could help distinguish antigen-side from execution-side progression at relapse and detect lineage drift before it becomes clinically overt. The second response is to reduce dependence on any single lineage-linked antigen through dual targeting. Because resistant cells may downregulate DLL3 while retaining other neuroendocrine surface markers (23), engaging a second antigen could maintain a targetable handle through a lineage shift, provided that the second antigen is not lost in the same transition. Candidates under study include B7-H3, SEZ6, TROP2, and other surface antigens enriched in SCLC (6–8). The deeper and still hypothetical version of this strategy is to act on plasticity itself. If induction rather than selection proves to drive lineage escape, modulating the transition from neuroendocrine to non-neuroendocrine states could in principle stabilize the antigen rather than chase it. However, this possibility depends on resolving the induction-versus-selection question. A concrete route to this idea is epigenetic co-targeting. Regulators such as EZH2 and LSD1 shape neuroendocrine lineage programs and Notch/ASCL1-linked plasticity, whereas MYC-driven NEUROD1-high or variant SCLC states show vulnerability to Aurora kinase inhibition (52–54). In the context of DLL3-directed redirection, these agents should therefore be viewed not simply as independent cytotoxic partners but as candidate tools to stabilize or constrain the lineage state that maintains DLL3 availability. This remains a hypothesis-generating strategy because the optimal direction of lineage modulation will depend on whether resistance is dominated by selection of pre-existing DLL3-low cells or active phenotypic induction.

### 6.2 Holding the execution axis: sustaining and protecting the effector

The execution axis fails when the recruited T cell cannot kill despite engagement. Here, strategies aim not at the antigen but at the durability and fitness of the effector response. The most direct approach is to build costimulation into the engager itself. In preclinical studies, DLL3-targeted trispecific molecules that add a 4-1BB (CD137) costimulatory signal to CD3 engagement increased intratumoral T-cell numbers and improved tumor control relative to conventional bispecifics in SCLC models. These molecules can be engineered so that costimulation is delivered only in the presence of DLL3-positive tumor cells, limiting systemic activation that would otherwise compound toxicity (50). The most clinically mature execution-axis combination pairs DLL3 redirection with PD-(L)1 blockade. The rationale is on-mechanism: redirected T-cell activation can induce adaptive checkpoint feedback that PD-(L)1 blockade is positioned to counter. In the phase 1b DeLLphi-303 study (NCT05361395), tarlatamab plus a PD-L1 inhibitor (atezolizumab or durvalumab) as first-line maintenance after chemo-immunotherapy showed manageable safety and encouraging antitumor activity, with a median overall survival of 25.3 months across 88 patients (51); this single-arm regimen is now being tested against PD-L1 maintenance alone in the randomized phase 3 DeLLphi-305 trial (NCT06211036). Combination experience nonetheless remains early, and the optimal sequencing, the safety of overlapping immune activation, and the patients most likely to benefit are unresolved.

A more radical execution-axis strategy substitutes the effector itself. Whereas tarlatamab redirects endogenous, polyclonal T cells transiently and off-the-shelf, DLL3-directed CAR-T cells supply an engineered, potentially self-renewing effector. DLL3-directed CAR-T approaches have entered early clinical testing, including the autologous DLL3 CAR-T product AMG 119 in relapsed/refractory SCLC (NCT03392064) (55). Preclinical work has extended the platform along two execution-axis-relevant lines: allogeneic DLL3 CAR-T cells that forgo patient-specific manufacturing and retain antitumor efficacy, including complete responses, in SCLC models (56), and cytokine-armored variants such as IL-18-secreting DLL3 CAR-T cells, which reduce exhaustion, promote a memory phenotype, and deepen responses, with additional benefit from PD-1 blockade (57). Both tarlatamab and DLL3-directed CAR-T cells recognize DLL3 independently of tumor-cell MHC and remain governed by the same two axes: loss of the lineage-defined antigen disables either, and both depend on successful immune execution. Their differences fall mainly on the execution axis. The transient pharmacology of a bispecific requires repeated dosing but imposes no manufacturing delay, an advantage in a disease that can progress too quickly for autologous cell production, whereas the persistence of a CAR product could in principle provide more durable surveillance. CAR-based therapy therefore does not escape the two-axis problem; it re-engineers the execution axis in the hope of holding it open longer.

A further set of approaches targets the niche rather than the effector, remodeling the immunosuppressive microenvironment through regulatory T-cell-supportive pathways, immunosuppressive myeloid populations, or myeloid checkpoint signals. These strategies address different points along the path from engagement to killing, but all share the aim of keeping the effector functional under the sustained activation imposed by redirection.

### 6.3 The two axes are coupled

Organizing strategies by axis is clarifying, but it should not imply that the axes can always be repaired in isolation, because they interact. Co-targeting a second antigen recruits more T cells, but it also subjects them to the same chronic stimulation that drives exhaustion. Costimulatory engagers that sustain T-cell activity will exert correspondingly greater selective pressure on antigen expression. A durable regimen therefore likely requires holding both axes at once: a stable or redundantly targeted antigen and a protected, non-exhausted effector. This is why monitoring that can distinguish the two failure modes at relapse is foundational rather than ancillary. The practical task is less to find a single better molecule than to keep two unstable systems aligned for longer.

## 7 Conclusion

DLL3-directed therapy is best understood not as a simple target-and-drug story but as an interface therapy acting at the junction between a plastic tumor lineage state and an activatable but potentially dysfunctional host immune system. The clinical trajectory of DLL3 illustrates that target validity alone does not determine therapeutic success; the platform-specific demands placed on the antigen and effector system are equally important. Rova-T showed that a DLL3-directed PBD-antibody-drug conjugate could achieve compelling preclinical activity and early clinical activity but failed to establish an adequate therapeutic index, whereas tarlatamab demonstrated that the same antigen can support survival-extending T-cell redirection. This contrast validates DLL3 as an actionable surface antigen without establishing that immune recognition is its only therapeutically viable use. Resistance correspondingly emerges when either system gives way: the lineage state may drift toward a DLL3-low or non-neuroendocrine identity, or the recruited effector may be functionally constrained before treatment or become exhausted under repeated activation. The lineage-defined vulnerability that the therapy exploits can, under the pressure of being exploited, double as the route of escape. The operative clinical question is therefore not only whether a tumor is DLL3-positive at baseline, but how stable the lineage state that presents DLL3 will prove under treatment, how fit the host effector compartment is before dosing, how durably it can execute after redirection, and whether both axes can be held in alignment long enough to convert response into durable benefit.
